# Anti-Brucella activity of *Caryopteris mongolica Bunge* root extract against *Brucella melitensis* infection in mice

**DOI:** 10.1186/s12906-018-2220-y

**Published:** 2018-05-03

**Authors:** Tsevelmaa N, Narangerel B, Odgerel O, Dariimaa D, Batkhuu J

**Affiliations:** 1 Core laboratory of Mongolian National University of Medical Sciences, Ulaanbaatar-26, Mongolia; 2 School of Engineering and Applied Sciences, National University of Mongolia, POB-617, Ulaanbaatar-46A, Mongolia; 3 Institute of Veterinary Medicine, Zaisan, POB 53 /24, Ulaanbaatar-17024, Mongolia

**Keywords:** Caryopteris mongolica Bunge, Brucella melitensis, In vivo anti-Brucella activity

## Abstract

**Background:**

The current treatment for human brucellosis requires a combination of antibiotics for long periods of time, and the reported incidence and prevalence of the disease vary widely in nomadic livestock of Mongolia. The objective of the present study was to evaluate the in vivo antibacterial activity of the *C. mongolica* root extract against *B. melitensis*.

**Methods:**

In this study, we used of 6 groups of mice (*n* = 5). Five groups of BALB/c mice were inoculated intraperitoneally with the M16 strain of *B. melintensis*, as follows: (i) one group was used for pretreatment monitoring; (ii) the control group was administered 2% Tween 80 and was used as the non-treatment group; and the other three groups were treated with one oral gavage per day for 21 days with (iii) doxycycline (2 mg/day), (iv) doxycycline (1 mg/day) with root extract (20 mg/day), and (v) *C. mongolica* root extract (20 mg/day). The one group that was kept non-infected was used as a healthy control group.

**Results:**

This study demonstrated that daily treatment with doxycycline alone and in combination with *C. mongolica* root extract significantly reduced splenic infection at the end of treatment. However, the spleen index of both the doxycycline-treated and the combination-treated groups of mice decreased by approximately 50% compared to that of the healthy control mouse group. Treatment with the *C. mongolica* root extract resulted in a 1.47log reduction in splenic infection compared to the non-treatment group, and the spleen index of the *C. mongolica*-treated group of mice was the same as that of the normal mouse group. In all treatment groups, neutrophil phagocytic activity significantly decreased, and all treatment groups demonstrated splenic regeneration.

**Conclusions:**

The present study showed that the *C. mongolica* root extract may be useful in the treatment of brucellosis patients, in combination with doxycycline or other antibiotics, to reduce the toxicity of high-dosage antibiotics, to prevent the development of antibiotic resistance and to prevent *Brucella* infection.

## Background

*Caryopteris mongolica* Bunge (Lamiaceae) grows in areas such as Khentii, Khangai, Mongol Daguur, Mongol Altai, and Dornod Govi in Mongolia and some areas of northern China. *C. mongolica* has long been used as a traditional medicine in Mongolia to alleviate aches, oedema, and rheumatism [1]. We have reported the phytochemical analysis of root extract of *Caryopteris mongolica*. We isolated of three new compounds and five known diterpene derivatives, namely, demethylcryptojaponol, incanone, 6α-hydroxydemethylcryptojaponol, cyrtophyllone B, and 14-deoxycoleon U, from the root extract of *Caryopteris mongolica,* used HPLC, MS and NMR techniques. Among these isolates, a new compound of abietane diterpene derivatives showed strong antibacterial activity [2]. *Brucella* is a gram-negative, aerobic, coccobacillary, non-motile microorganism [3]. In addition, *Brucella* species are facultative intracellular pathogens that localize predominantly in the cells and organs of the mononuclear phagocytic system, such as macrophages in the liver and spleen [4]. Brucellosis mainly affects domestic animals, in which *Brucella* colonize the reticulendothelial system and genital organs, leading to abortion, stillbirth, orchitis, epididymitis, and infertility and resulting in significant economic losses. The illness continues to be one of the most widely distributed zoonoses and is transmissible to humans [5, 6]. Human brucellosis is usually caused by *Brucella melintensis* and has a wide spectrum of clinical symptoms, including irregular fever, sweating, arthralgia, myalgia, headache and weakness [7]. The reported incidence and prevalence of the disease vary widely in nomadic livestock in Mongolia. Selenge T., and others reported that 20.2%, or one in five, herders are infected with brucellosis [8]. The current treatment for human brucellosis requires a combination of antibiotics for long periods of time because antibiotics either lose their antimicrobial activity in the intracellular environment or do not persist long enough to produce a therapeutic effect [9]. World Health Organization guidelines recommend a 6-week course of doxycycline plus rifampin [10]. More recent recommendations also propose the use of doxycycline for 6 weeks with the aminoglycoside streptomycin for 2 to 3 weeks or with gentamicin for 1 week [11]. Combination therapies are more effective than single-agent therapies; however, new therapies are necessary due to the difficulties of patient adherence to the treatment itself along with the side effects of combination therapy and the dangers of antibiotic resistance [12]. Herbal treatments are gentle, inexpensive and effective in controlling the disease. On the basis of the in vitro screening results, the *C. mongolica* root extract had high anti-*Brucella* activity against *B. melintensis* (a concentration of 50 μg/disc produced an inhibition zone of 15 mm). To identify the anti-*Brucella* activity of the crude extract of *C. mongolica* root, we used the disc diffusion method. *B. melitensis* was grown in tryptone soy broth media for 72 h, and 100 μl (10^6^ CFU/ml) was spread over the surface of tryptone soy agar medium in Petri dishes with diffusion discs treated with the root extract. Doxycycline-treated discs (a concentration of 10 μg/disc produced an inhibition zone of 35 mm) were used as a positive control. In other studies, plants such as *Scrophularia deserti* [13], *Origanum syriacum, Thymus syriacus* [14], *Prunus mahaleb seeds* [15], *Satureja hortensis* [16], *Oliveria decumbens* [17], *Teucrium polium* [18], and *Moringa oleifera* [19] have been examined for in vitro activity against *Brucella*. However, studies have not reported the efficacy of these medicinal plants for the in vivo treatment of brucellosis. The aim of this study was to evaluate the in vivo antibacterial activity of the *C. mongolica* root extract against *B. melitensis*.

## Methods

### Bacterial strain and culture conditions

Tryptone soy agar (TSA), Brucella agar, and tryptone soy broth (TSB) were purchased from Biolab, Hungary. *Brucella melintensis* 16 M (ATCC23456, biotype 1), a smooth virulent strain, was used in this study. The experiment was performed with fresh bacteria incubated on TSA plates at 37 °C until the exponential growth phase. The bacterial clones were then counted and diluted with sterile saline solution.

### Plant material

The roots of *Caryopteris mongolica* were collected in August 2014 from the Bayanchandmani soum of Tov Province. The plant was identified by Dr. Ch. Sanchir of the Institute of Botany at the Mongolian Academy of Sciences. A voucher specimen has been deposited in the herbarium at the National University of Mongolia (No. 20140803).

The *C. mongolica* root was extracted with acetone:H_2_O (8:2), evaporated, and air dried. For the in vivo study, the dry extract was dissolved in Tween 80 (polyoxyethylene [20] sorbitan monooleate), which was purchased from Sigma. We confirmed the acute toxicity of the *C. mongolica* root extract in accordance with Guideline 423 of the Globallу Harmonised System. The solution of dried root extract in Tween 80 was administered by oral gavage at concentrations of 1000, 3000, 5000, 8000, and 15,000 mg/kg to 3 mice per concentration. The results of this experiment showed that *C. mongolica* root would be classified as non-toxic (data not shown).

### Animals

Female BALB/c mice of 12–15 weeks of age (weight 25–28 g) were supplied by the Research Institute of Veterinary Medicine. The mice were randomly placed into 6 groups (*n* = 5 mice/group), housed in transparent cages and fed a normal laboratory diet of nutrient pellets with access to drinking water. The mice were housed in a temperature-controlled facility (23 ± 5 °C) with a 12 h light-dark cycle. All experimental protocols were approved by the Animal Care and Ethics Committee of the Institute of Veterinary Medicine of Mongolia.

### Inoculation

The mice were infected with a single intraperitoneal dose of 1.7 × 10^5^ CFU/mouse *Brucella melintensis* in 0.1 ml of sterile 0.9% saline. The one group that was kept non-infected was used as a healthy control group. On the 15th day after infection, a group of mice was selected to have their spleens aseptically removed and examined for bacterial infection.

### Treatment

The control group (ii) was kept untreated (administered 2% Tween80 solution) and was sacrificed at 38 days post-infection. The other groups were administered a daily oral gavage for 21 days, as follows: (iii) antibiotic only, (iv) antibiotic and extract, and (v) extract only. The respective doses for each mouse in these groups were as follows: 2 mg doxycycline in 0.1 ml (80 mg/kg/day), 1 mg doxycycline + 20 mg extract in a total of 0.3 ml, and 20 mg extract in 0.25 ml (800 mg/kg/day) at intervals of 24 h. Doxycycline was purchased from Sigma Aldrich for use as an antibacterial treatment.

### Bacterial counts in spleen

At the end of the treatment period (38 days post-infection), we first collected blood samples from the tail vein of the mice in all groups and then aseptically removed the spleens and placed them inside pre-weighted sterile plastic Whirl-Pak Bags (Nasco) for weighing. The mice were anaesthetized with isoflurane delivered by an RC^2^-rodent Circuit Controller (VetEquip, California, USA) and euthanized in a CO_2_ chamber. To the sterile plastic bag containing each spleen, we added a solution of 9 parts sterile saline and then homogenized the tissue by hand. After the samples were homogenized, 10-fold serial dilutions of the homogenate were performed with a sterile saline solution; 100 μl of the dilutions was then inoculated onto TSA plates. The inoculum was distributed with a sterile spatula until fully dispersed on the agar surface. Two plates per sample were inoculated. The plates were incubated at 37 °C for 3 days. The splenic bacterial loads were calculated by multiplying the CFU by the dilution factor. The spleen index is a comparison of the spleen weight with the body weight. The bacterial counting experiments were conducted in accordance with the protocol described by Elias B et al. [20].

### Haemotological and histological analysis

The blood samples were collected from the tail vein of anaesthetized mice into heparinized tubes and were analysed with a blood analyser (Diff/poch-100ivd Sysmex, Japan). Additionally, neutrophil phagocytic activity was examined by the nitroblue tetrazolium (NBT) reduction test (Sigma Aldrich procedure number 840). For the histological assay, the spleens were fixed in a 10% formalin solution for 24 h; washed in flowing water; dehydrated in ethanol, xylene, and paraffin solutions; and then embedded in paraffin. The paraffin blocks were sliced with a Yamato Konki microtome and stained with haematoxylin-eosin (HE). Finally, the slides were analysed with a Nikon E-600 microscope.

### Statistical analysis

One-way ANOVA followed by the Newman–Keuls multiple comparison test was used to compare the results between the samples using GraphPad Prism 5 (*P* ≤ 0.05 or 0.01).

## Results

### Comparison of infection-related physical and splenic changes in treatment groups

**Fig. 1:**
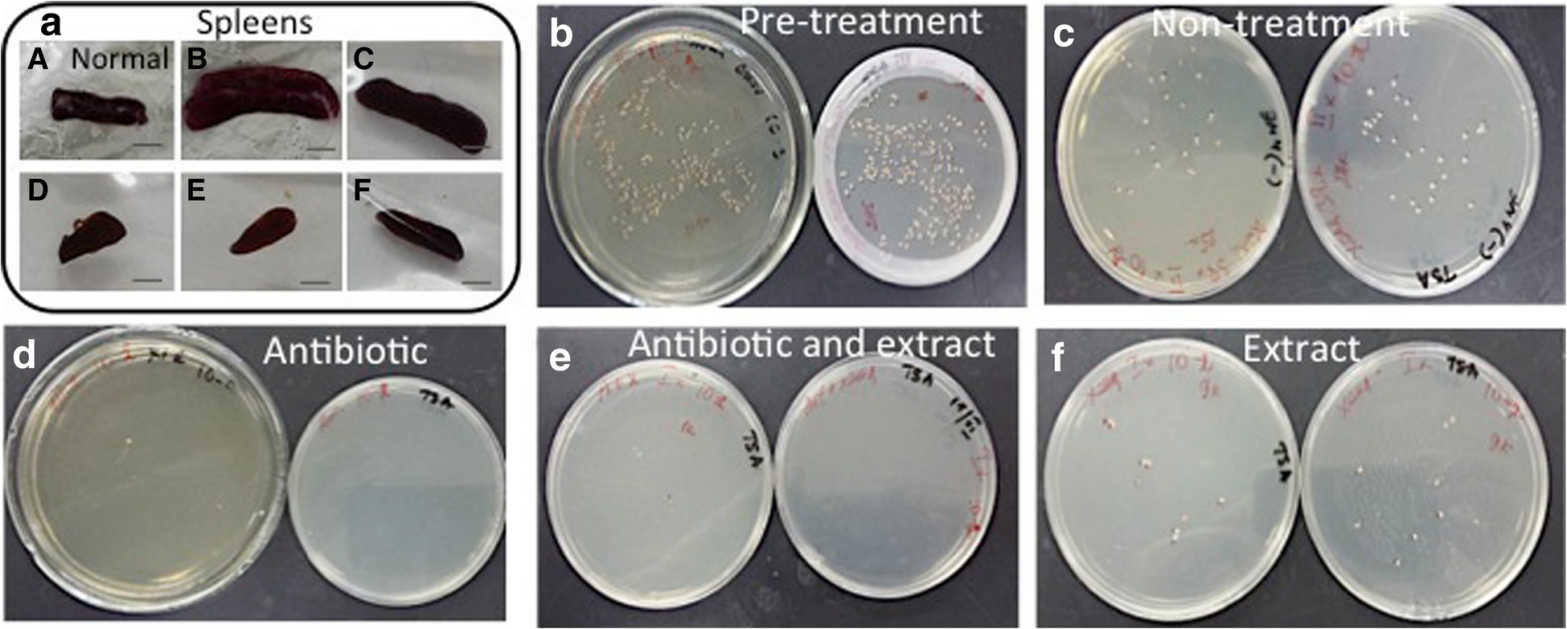
Splenic enlargement in a *Brucella*-infected mouse compared with a normal mouse and countable *Brucella* colonies in 0.1 g spleen homogenate from mice in the following groups inoculated on TSA plates. **a** health control mouse spleen, **b** pretreatment, **c** non-treatment, **d** doxycycline only, **e** combination of antibiotic and root extract, **f** *C. mongolica* root extract only

We first examined splenic infection on the 15th day post-infection. The spleen index of the pretreatment (post-infection day 15) monitoring group (i) was increased threefold (Table 1 and Fig. 1b) compared to that of the normal mouse group.

**Table 1:** Antibacterial efficacies of *C. mongolica* root extract, antibiotic, and the combination of both treatments in *B. melitensis*-infected BALB/c mice

Treatment group	Spleen index (mean ± SD)	Spleen index reduction	Log CFU in 0.1 g spleen (mean ± SD)	Log reduction
Non infected	0.3 ± 0.2	Control	–	
Pretreatment	1.09 ± 0.4*	↑0.79	5.46 ± 0.15	
Non treatment	0.96 ± 0.2*	↑0.66	4.5 ± 0.1	Control
Doxycycline	0.15 ± 0.01*	↓0.15	2.05 ± 0.07**	2.45
Combination treatment	0.19 ± 0.07*	↓0.11	2.1 ± 0.2**	2.4
*C.mongolica* root extract	0.32 ± 0.05	↑0.02	3.03 ± 0.15	1.47

Statistical analysis: *P < 0.05 and **P < 0.01 comparing the non-infected and non-treatment groups

In addition, the administered *Brucella* inoculum produced a baseline splenic infection of 5.46 log CFU/0.1 g spleen (Table 1 and Fig. 1b). This result showed that *B. melitensis* infection was successful in the mice. We started treatment with antibiotic, *C. mongolica* root extract or a combination of the two 15 days after inoculation to allow the establishment of chronic infection.

Within the first 10 days of treatment, the mice in the groups that were administered antibiotic and the combination of antibiotic and root extract displayed physical signs of weakness, slower body movement, and ruffled fur. Furthermore, 2 mice in the group treated with only antibiotic died before the end of treatment. However, these signs associated with ill health were not evident in the group that was administered the root extract treatment.

At the end of treatment, we examined splenic infection in the non-treatment (day 38 post-infection) control group (ii) and observed that the bacteria were gradually eliminated compared to the pretreatment group. The mean splenic bacterial count in the non-treatment group was 4.5 log CFU (Table 1, Fig. 1c). Thus, the treatment efficacies in the treated groups were compared to the non-treatment group by the splenic bacterial count.

In comparison with no treatment, daily treatment with doxycycline alone (iii) (2.05 log CFU) and with the combination of doxycycline and *C. mongolica* root extract (iv) (2.1 log CFU) significantly reduced splenic infection at the end of treatment. However, the spleen index of both the doxycycline-treated and the combination-treated mouse groups decreased by approximately 50% compared to that of the normal mouse group (Table 1, Fig. 1a, d, e). The *C. mongolica* root extract-treated group (v) demonstrated a 1.47 log CFU reduction in splenic bacterial count compared with the non-treatment group, for which the spleen index was the same as that of the normal mouse group (vi) (Table 1, Fig. 1a, f). We included 5 mice in each group; however, we used 3 mice from the antibiotic treatment group for the spleen index calculation. Furthermore, we used 3 mice from each group except the healthy control group for the splenic CFU counting experiment.

### Comparison of haematological changes in treatment groups

We also examined the haematological parameters of *B. melitensis*-infected BALB/c mice that were treated with *C. mongolica* root extract, antibiotic or a combination of both. The white blood cell (WBC) count of the mice in the pretreatment and non-treatment groups, compared to that of the normal mice, increased twofold. On the other hand, the WBC count decreased onefold in all post-treatment groups compared to the WBC count of the non-treatment group. However, the counts of other cell types (LYM, OTHR, EO) showed no significant reduction in any post-treatment group compared to those of the non-treatment group. Neutrophil phagocytic activity increased about threefold in the non-treatment group (at 38 days post-infection) compared to the normal group; in contrast, it decreased significantly in all treatment groups compared to the control group. In the normal mice, the neutrophil phagocytic activity was 13%, and it increased to 38% in the non-treatment group. Neutrophil phagocytic activity decreased to 14.5, 18.5 and 21% in the groups administered antibiotic only, combination therapy and extract only, respectively (Table 2). We included 5 mice in each group; however, we used 3 mice from the antibiotic treatment group for blood analysis.

**Table 2:** Haematological parameters of *B. melitensis*-infected BALB/c mice treated with *C. mongolica* root extract, antibiotic or the combination of both

Treatment group		Content of Blood cells (in μl)			
	WBC (×10^3^)	LYM (%)	OTHR (%)	EO (%)	Neutrophil phagacytosis activity (%)
Non infected	3.05 ± 0.2	73.4 ± 8.5	22.2 ± 4	1.4 ± 0.28	13 ± 2.8
Pretreatment	6.4 ± 0.7	59.7 ± 4.2	38.03 ± 5.6	3.6 ± 0.75	49 ± 6.5
Non treatment	5.7 ± 0.1	63.8 ± 9.02	34.9 ± 8.9	1.26 ± 0.7	38 ± 2
Doxycycline	4.7 ± 0.01*	60.05 ± 0.3	37.5 ± 0.3	2.4 ± 0.1	14.5 ± 3.5*
Combination treatment	4.3 ± 0.5*	61.8 ± 8.4	32.7 ± 5.5	1.93 ± 0.4	18.5 ± 3.5*
*C.mongolica* root extract	4.2 ± 0.9*	63.4 ± 3.6	34.4 ± 3.3	2.1 ± 0.9	21 ± 2.5*

Statistical analysis: *P < 0.05 compared to the non-treatment group

### Comparison of histological changes in treatment groups

**Fig. 2:**
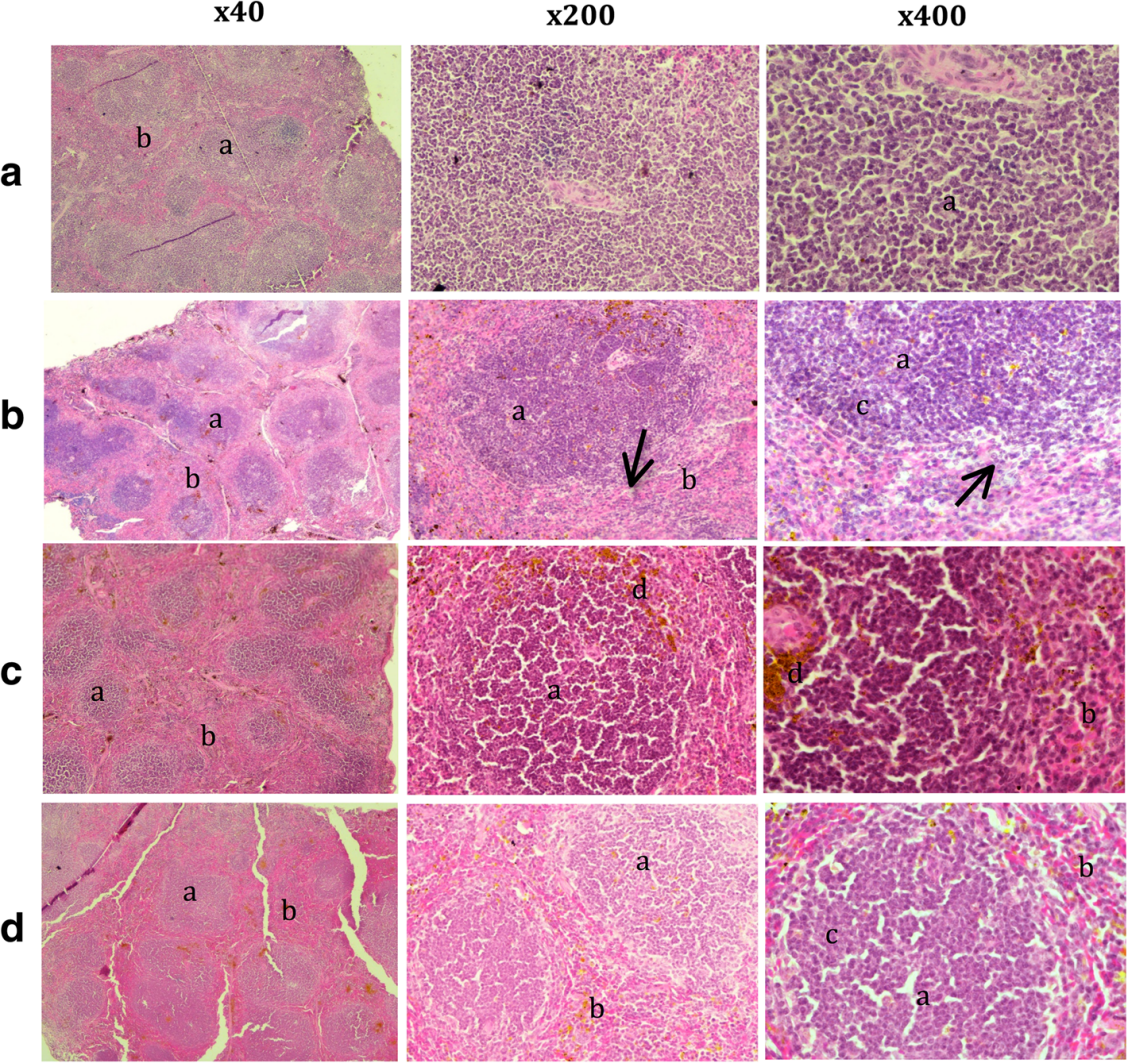
Splenic section obtained after administration in mice from the following groups: **a** normal, **b** non-treatment, **c** combination of antibiotic and root extract, **d** *C. mongolica* root extract only. *a* - white pulp, *b* - red pulp, *c* - lymphocytes in different stages of development, *d* - haemosiderin pigmentation, ↗ - lymphocyte necrosis. Haematoxylin and eosin stain, magnification 400× and 40×

The splenic histological study showed that lymphocyte necrosis occurred around the white pulp and increased the amount of red pulp, lymphocytes at different stages of development, macrophages and neutrophils in the non-treatment group compared with other groups (Fig. 2a, b). On the other hand, the mouse groups that were administered the combination of antibiotic plus root extract and root extract only exhibited a decrease in red pulp and neutrophils, as well as regeneration of areas of splenic necrosis (Fig. 2c, d). Interestingly, in the combination-treated group, haemosiderin pigmentation at the interface of the red and white pulp can be observed (Fig. 2c). We used of 2 mice from the healthy control, non-treatment, combination and only *C. mongolica* root extract treatment groups for the histological experiment.

## Discussion

The efficacy of different antibiotics for the treatment of human brucellosis has also been evaluated in mouse models [20–22]. Sasha and coworkers reported that mice treated with doxycycline (80 mg/kg) by the oral route demonstrated a reduction in bacterial count of 3.3 log CFU compared to control mice, whereas mice treated with ciprofloxacin by the subcutaneous (40 mg/kg), digestive (200 mg/kg), or intraperitoneal (20 mg/kg) routes are not able to control the infection [21, 23]. Our study demonstrated that the doxycycline-treated and combination dosage-treated groups showed a reduction of 2.4 log CFU and the group treated only with root extract demonstrated a reduction of 1.47 log CFU compared to the control group. Interestingly, in the doxycycline-treated and combination-treated groups, spleen sizes were dramatically reduced (a decrease of approximately 1.5 times) compared to the spleen sizes of the normal mice. The group treated only with *C. mongolica* root extract displayed more modest results in the ability to kill *Brucella* compared to the other groups; however, the spleen sizes of the mice in this group were the most similar to the spleen sizes of normal mice. Additionally, in the combination-treated group, haemosiderin pigmentation at the interface of the red and white pulp can be observed.

Haemosiderin is a golden-brown, granular pigment derived from the breakdown of red blood cells and is present within the cytoplasm of macrophages. Background haemosiderin levels reflect the normal removal of effete red blood cells by the spleen, but the haemosiderin level can increase in cases of chemically induced haemolytic anaemia or methaemoglobinaemia [24]. Rare side effects of doxycycline include haemolytic anaemia, thrombocytopaenia, eosinophilia, and neutropaenia [25–27]. Several studies have demonstrated that histological examinations reveal characteristic aminoglycoside-induced renal alterations such as tubular lipidosis and tubulonephrosis foci in mice that have received a gentamicin solution either alone or in combination with doxycycline [28, 29].

We predicted that a significant effect on spleen size resulting from the strong antibiotic treatment could cause anaemia to occur. Anaemia may lead the mice in the antibiotic-treated or combination-treated groups to exhibit physical signs of discomfort, such as motor weakness and ruffled fur. Root extract has been seen as a gentler treatment alternative, with a therapeutic effect that has yet to produce abnormal symptoms such as a reduction in spleen size. Previous histological studies note that the white pulp/red pulp ratio in the normal spleen is close to 1/1, while the ratio in the brucellosis-infected spleen is close to 1/4 [30]. *Brucella* bacteria are removed from the local tissue lymphocytes and transferred into the general circulation, from whence they are disseminated throughout the body, with a characteristic tropism for the cells and organs of the mononuclear phagocytic system, such as macrophages in the liver and spleen [31]. After two weeks of infection, concomitant with splenic swelling, granulomas and giant cells increase in this organ; a trend that continues until the middle of the chronic steady phase. During the 3rd week post-infection, the apparent depletion of lymphocytes in the white pulp reaches its maximum. Then, after the 4th week, lymphoid hyperplasia and extra medullary haematopoiesis gradually increase, with the appearance of several mitotic figures and the multifocal accumulation of macrophages that surround and sometimes cover the periarteriolar lymphoid sheaths. Therefore, the infiltration of macrophages and neutrophils increases their number to relatively large proportions. This increase is proportional to the swelling and infiltration of blood and phagocytic cells in the spleen [32]. Our study noted an increase in red pulp, smeared splenic cells and a high activity of neutrophil phagocytosis in the mice in the non-treatment group compared to those in the treatment groups. Mice in the *C. mongolica* root extract-treated group demonstrated regeneration of areas of splenic necrosis and a normal ratio of white to red pulp. Neutrophil phagocytic activity increases in the presence of bacterial infection, whereas the effectiveness of antibiotic therapy may be indicated by a reduction in the percentage of neutrophils positive for phagocytosis. The mean baseline neutrophil phagocytic activity was 9.6% in normal dogs, and a two- to fourfold increase was demonstrated in endotoxin-stimulated dogs [33, 34].

The haematological and histological results clearly showed that the *C. mongolica* root extract is effective for curbing inflammatory processes in *B. melitensis*-infected mice.

The antibiotics currently used for the treatment of human brucellosis are capable of acting efficiently under acidic conditions because they have to penetrate macrophages. Celli Jet al. indicated that Brucella organisms integrate with components of the early endosomal pathway and reside in acidified phagosomes but not in lysosomes [35]. Interestingly, gentamicin modified into hydrophobic and non-capsulated forms enhanced the intracellular killing of Brucella, thereby decreasing intracellular infection [36]. Doxycycline, like other tetracycline antibiotics, is bacteriostatic activity. Doxycycline works by preventing bacteria from reproducing through the inhibition of protein synthesis. The lipophilic properties of doxycycline may account its ability to inhibit some bacterial strains that are more hydrophilic agents [37]. Our previous study demonstrated of three new compounds and five known diterpene derivatives from the root extract of *C. mongolica*. Among these isolates, a new compound of abietane diterpene derivatives showed strong antibacterial activity [2]. The diterpenoids can act on multiple biochemical targets of the bacteria and it has been suggested that the activity of these compounds results from their ability to cross or damage microbial cell membranes due to their amphiphilic property [38, 39]. Urzua *et al.* suggested that promote the efficient antibacterial effect displayed by diterpenoids include a lipophilic decalin ring system, capable of insertion into a lipophilic region, and one strategically positioned hydrogen-bond-donor group (HBD; hydrophilic group), capable of interactions with the membrane phosphorylated groups. These hydrogen-bond-interactions between the phosphorylated groups in the membrane and the HBD in the diterpene are very important for the antibacterial activity. The totarol diterpene inhibits NADH-CoQ in the bacterial electron transport chain [40]. Horminone, an abietane diterpene quinone inhibiting bacterial protein synthesis, according bind to ribosomal RNA phosphate groups [41], ferruginol potentiates activity by acting as an efflux pump inhibitor [42]. The quinone diterpenoids of *C.mongolica* root may obtainable penetrate into the lipophilic cell membrane and bind to phosphate groups on macromolecules due to this, degrading of the cell membrane or inhibiting of proteins synthesis.

The recently study validated of pharmacokinetics of three diterpenes (carnosic acid, carnosol, rosmanol) after oral administration in rats of different doses (0.25, 0.82, 2.45 g/kg) *Rosemarine officinalis*.L extracts. They suggested that diterpenes are rapidly absorb, slowly eliminate, the values of maximum plasma concentration (*C*_max_), the time to reach the maximum concentrations (*t*_max_) directly depend on dosage. In the elimination phase, a double-peak phenomenon appeared on the mean plasma concentration-time profiles. The probably reason may be caused by redistribution and enterohepatic circulation after the analytes were excreted into the gastrointestinal tract through the bile [43]. Our previous study demonstrated that diterpenes are main components which antibacterial active in *C.mongolica* root extract. Also, *R.officinalis* and *C.mongolica* are both belong in *Lamiaceae* family. Therefore pharmacokinetic properties of *C.mongolica* extract may similar with the suggested study. Further, its necessary of action mechanism and pharmacokinetic study for new diterpenoids from *C.mongolica* root. Also, we have to more study about synergistic or additive effect of *C.mongolica* extract with other anti-brucella agents.

This study clearly demonstrated that crude extracts from *C. mongolica* root have good efficacy against in vivo *B. melitensis* infection and “mild” or “low toxicity” effective than antibiotic treatment.

## Conclusions

This study demonstrated that daily treatment with doxycycline both alone and in combination with *C. mongolica* root extract significantly reduced splenic infection at the end of the treatment. However, the spleen index of both the doxycycline-treated and the combination-treated mouse groups decreased by approximately 50% compared to that of the normal mice group. The *C. mongolica* root extract-treated group demonstrated a 1.47 log CFU reduction in splenic infection compared to the non-treatment group and a spleen index that was similar to that of the normal mouse group. In all treatment groups, the activity of neutrophil phagocytosis significantly decreased, and splenic regeneration was observed. The present study has concluded that *C. mongolica* root extract may be useful in the treatment of brucellosis patients, in combination with doxycycline or other antibiotics, to avoid the toxicity of high dosage antibiotics and prevent the development of antibiotic resistance. Furthermore, the root extract can possibly be used to prevent *Brucella* infection.

## References

[1] Batkhuu J, Sanchir C, Ligaa U, Jamsran T (2005). Colored illustrations of Mongolian useful plants.

[2] Saruul E, Murata T, Selenge E, Sasaki K, Yoshizaki F, Batkhuu J (2015). An antibacterial ortho-quinonediterpenoid and its derivatives from *Caryopteris mongolica*. Bioorg Med Chem Lett.

[3] Doganay M, Aygen B (2003). Human brucellosis:an overveiw. Int J Infect Dis.

[4] Pappas G, Papadimitriou P, Akritidis N, Christou L, Tsianos EV (2006). The new global map of human brucellosis. Lancet Infect Dis.

[5] Xavier MN, Costa EA, Paixao TA, Santos RL. The genus *Brucella* and clinical manifestations of brucellosis. Ciencia Rural. 2009;(7):2252–60.

[6] Thoen CO, Enright F, Cheville NF (1993). *Brucella* in Pathogenesis of Bacterial Infections in Animals.

[7] McLean DR, Russell N, Khan MY (1992). Neurobrucellosis: clinical and therapeutic features. Clin Infect Dis.

[8] Ts S, Ts N, Zolzaya B, Gantsetseg D (2010). diagnosis for spread of brucellosis. Mongolian J Infectious Disease.

[9] Gamazo C, Lecaroz MC, Prior S, Vitas AI, Campanero MA, Irache JM, Blanco-Prieto MJ (2006). Chemical and biological factors in the control of Brucella and brucellosis. Curr Drug Deliv.

[10] World Health Organization Joint FAO/WHO Expert Committee on Brucellosis Sixth report (1986). World health organ. Tech. Rep. Ser.

[11] Ariza J, Bosilkovski M, Cascio A, Colmenero JD, Corbel MJ, Falagas ME, Memish ZA, Roushan MR, Rubinstein E, Sipsas NV, Solera J, Young EJ, Pappas G (2007). Perspectives for the treatment of brucellosis in the 21st century the Ioannina recommendations. PLoS Med.

[12] Pappas G, Solera J, Akritidis N, Tsianos E (2005). New approaches to the antibiotic treatment of brucellosis. Int J Antimicrob Agents.

[13] Pouya P, Mahmoud R, Hassan M, Fariba B, Sara SM, Mahmoud B (2012). Study of the ethanol extract of *Scrophularia deserti* effect on Brucella melitensis in comparison with streptomycin. Asian Pac J Trop Biomed.

[14] Ayman A, Mazen S (2013). The antibacterial activity of selected labiatae (*Lamiaceae*) essential oils against *Brucella melintensis*. Iran J Med Sci.

[15] Seyyednejad SM, Maleki S, Mirzaei Damabi N, Motamedi H (2008). Antibacterial activity of *Prunus mahaleb* and parsley (*Petroselinum crispum*) against some pathogen. Asian J Biol Sci.

[16] Adiguzel A, Ozer H, Kilic H, Cetin B (2007). Screening of antimicrobial activity of essential oil and methanol extract of *Satureja hortensis* on food borne bacteria and fungi. Czech J Food Sci.

[17] Motamedi H, Darabpour E, Gholipour M, Seyyed Nejad SM (2010). In vitro assay for the anti-Brucella activity of medicinal plants against tetracycline-resistant *Brucella melitensis*. J Zhejiang Univ Sci B.

[18] Seyyednejad SM, Motamedi H (2010). A review on native medicinal plants in Khuzestan, Iran with antibacterial properties. Int J Pharmacol.

[19] Alawad MFEM (2012). Antibrucella activity of *Moringa oleifera in vitro* against *Brucella abortus*. Int J Inf Dis.

[20] Elias BC, Carlos C, Esbetan C, Edgardo M (2013). Bacterial counts in spleen. Bioprotocol.

[21] Shasha B, Lang R, Rubinstein E. Therapy of experimental murine brucellosis with streptomycin, co- trimoxazole, ciprofloxacin, ofloxacin, pefloxacin, doxycycline, and rifampin. Antimicrob Agents Chemother. 1992;(5):973–6. 10.1128/aac.36.5.973 PMC188798 1510422

[22] Prior S, Gander B, Irache JM, Gamazo C. Gentamicin loaded microspheres for treatment of experimental Brucella abortus infection in mice. J Antimicrob Chemother. 2005;(6):1032–6. 10.1093/jac/dki144 15883176

[23] Atkins HS, Spencer S, Brew SD, et al. Efficacy of ciprofloxacin versus doxycycline as prophylaxis against experimental murine *Brucella melitensis* infection. Int J Antimicrob Agents. 2009;(5):474–6. 10.1016/j.ijantimicag.2009.04.006 19500948

[24] Travlos GS, Morris RW, Elwell MR, Duke A, Resenblum S, Thompson MB (1996). Frequency and relationships of clinical chemistry and liver and kidney histopathology findings in 13-week toxicity studies in rats. Toxicology.

[25] Sloan B, Scheinfeld N (2008). The use and safety of doxycycline hyclate and other second-generation tetracyclines. Expert Opin Drug Saf.

[26] Del Rosso JQ (2006). Systemic therapy for rosacea: focus on oral antibiotic therapy and safety. Cutis.

[27] Simpson MB, Pryzbylik J, Innis B, Denham MA (1985). Hemolytic anemia after tetracycline therapy. N Engl J Med.

[28] Mingeot-Leclercq MP, Tulkens PM (1999). Aminoglycosides: Nephrotoxicity. Antimicrob Agents Chemother.

[29] Nagai J, Takano M (2004). Molecular aspects of renal handling of amino-glycosides and strategies for preventing the nephrotoxicity. Drug Metab Pharmacokinet.

[30] Charting A New Course in Tissue Analysis http://www.flagshipbio.com/uncategorized/the-spleen-the-whole-spleen-and-nothing-but-the-spleen/.

[31] Murphy EA, Sathiyaseelan J, Parent MA, Zou B, Baldwin CL (2001). Interferon- gamma is crucial for surviving a Brucella abortus infection in both resistant C57BL/6 and susceptible BALB/c mice. Immunology.

[32] Murphy EA, Parent M, Sathiyaseelan J, Jiang X, Baldwin CL (2001). Immune control of Brucella abortus 2308 infections in BALB/c mice. FEMS Immunol Med Microbiol.

[33] Douwes FR (1972). Clinical value of NBT test. N Engl J Med.

[34] John WH, Wilson JW (1973). Nitroblue tetrazolium reduction by neutrophils in experimental hemorrhagic shock. Am J Pathol.

[35] Celli J, Chastellier C, Franchini DM, Pizarro-Cerda J, Moreno E, Gorvel JP (2003). Brucella evades macrophage killing via VirB-dependent sustained interactions with the endoplasmic reticulum. J Exp Med.

[36] Edurne I, Carlos G, Hugo L, Miguel AC, David S, Ana GG, Elisa E (2013). Hydrophobic gentamicin-loaded nanoparticles are effective against *Brucella melitensis* infection in mice. Antimicrob Agents Chemother.

[37] Eric MS, William BP. Antimicrobial drugs. Oxford press. 2000:187.

[38] Urzua A, Rezende M, Mascayano C, Vásquez L (2008). A structure-activity study of antibacterial diterpenoids. Molecules.

[39] Simoes M, Bennett RN, Rosa EAS (2009). Understanding antimicrobial activities of phytochemicals against multidrug resistant bacteria and biofilms. Nat Prod Rep.

[40] Haraguchi H, Oike S, Muroi H, Kubo I (1996). Mode of action of totarol, a diterpene from Podocarpus nagi. Planta Med.

[41] Nicolás I, Vilchis M, Aragón N, Miranda R, Hojer G, Castro M (2003). Theoretical study of the structure and antimicrobial activity of horminone. Int J Quantum Chem.

[42] Smith ECJ, Williamson EM, Wareham N, Kaatz GW, Gibbons S (2007). Antibacterials and modulators of bacterial resistance from the immature cones of Chamaecyparis lawsoniana. Phytochemistry.

[43] Liqian W, Chunli G, Zhibin W, Lu L, Mingjie G, Qian L, Chunjuan Y (2017). Determination and pharmacokinetic study of three diterpenes in rat plasma by UHPLC-ESI-MS/MS after oral administration of *Rosmarinus officinalis* L. extract. Molecules.

